# Qualitative and Quantitative Proteomic Analysis of Venoms from Mexican Rattlesnakes

**DOI:** 10.3390/toxins18060256

**Published:** 2026-06-05

**Authors:** Lizbeth Hernández-Ancheyta, Víctor Hugo Reynoso, Juan Carlos López-Vidal, Javier Hernández-Sánchez, Karen Delgadillo-Gutiérrez, María Lilia Domínguez-López, Julieta Luna-Herrera

**Affiliations:** 1 Departamento de Inmunología, Escuela Nacional de Ciencias Biológicas, Instituto Politécnico Nacional, Ciudad de México 11340, Mexico; lizbeth.heran@gmail.com (L.H.-A.); kdelgadillog@ipn.mx (K.D.-G.); mdominguezl@ipn.mx (M.L.D.-L.); 2 Departamento de Zoología/Pabellón Nacional de la Biodiversidad, Instituto de Biología, Universidad Nacional Autónoma de México, Ciudad de México 04510, Mexico; 3 Departamento de Zoología, Escuela Nacional de Ciencias Biológicas, Instituto Politécnico Nacional, Ciudad de México 11340, Mexico; 4 Departamento de Genética y Biología Molecular, Centro de Investigación y de Estudios Avanzados del Instituto Politécnico Nacional, Ciudad de México 07360, Mexico; 5 Secretaría de Ciencia, Humanidades, Tecnología e Innovación (SECIHTI), Ciudad de México 03940, Mexico

**Keywords:** *Crotalus*, snake venomics, quantitative proteomics, venom phylogeny

## Abstract

Despite the vast biodiversity of Mexican vipers, venom of endemic species has been barely studied. Here we analyzed the venom composition of three endemic species of rattlesnakes: *Crotalus aquilus*, *C. triseriatus*, and *C. ravus*. We used quantitative chromato-mass-spectrometry and compared venoms with *C. molossus*, a species commonly found in North America, in a comparative and phylogenetic framework. In total, we identified 165 proteins grouped in 19 main protein families, consistent with previous reports for viperid venoms. In *C. aquilus* and *C. triseriatus*, the most predominant protein-family type was Serine Proteases, and in *C. triseriatus* and *C. molossus* it was Snake Venom Metalloproteases. The Label-free quantification revealed a high proportion of Snake Venom Metalloproteases in *C. aquilus*, *C. triseriatus*, and *C. molossus*, reaching 28–47% of the total venom. In contrast, in *C. ravus* 47% of the venom was composed of Phospholipases A_2_. Among the four species analyzed, *C. triseriatus* and *C. aquilus* were most similar in compositional profiles and their profiles are highly correlated. Venom composition in terminal clades and taxa were better explained by protein losses than evolution of new proteins. The *triseriatus* group share seven proteins, while the clade *C. aquilus* + *C. triseriatus* share seven derived protein features, of which six are protein losses.

## 1. Introduction

The World Health Organization (WHO) considers snakebite envenoming as a public health problem in developing countries, causing around 81,000 to 138,0000 deaths and 400,000 permanent disabilities each year [1]. In the American continent, Mexico ranked second as the country with most deaths caused by venomous snake bites per year [2]. Mexico is also the second country with the most venomous snake species worldwide, most of them endemic. However, the knowledge of venom composition of most species remains unknown. Only some species have been studied in the last few years [3,4,5,6,7,8,9]. *Crotalus* spp. and *Bothrops* spp. represent relevant groups for public health services due to the consequences of bites [10,11,12].

Venom of *Crotalus* species has both myotoxic and neurotoxic effects [11,12,13]. Symptoms of human envenomation include a variety of local and systemic effects such as pain in the bite area, swelling, numbness, hypotension, hemorrhages, coagulation disorders, and muscle damage [10]. Clinically relevant protein families frequently reported within *Crotalus* species are Phospholipases A_2_ (PLA_2_), Snake Venom Metalloproteases (SVMPs) and Serine Proteases (SPs) [6,9,14,15,16,17].

PLA_2_ can be found in all venomous snake taxa (elapids and viperids), causing a broad spectrum of pharmacological and biological effects due to its neurotoxic, hemolytic myonecrotic, anticoagulant, hypotensive, and edema-inducing activities [18,19]. Neurotoxic PLA_2_, associated with the high toxicity of the venom, has been reported in *C. scutulatus*, *C. viridis*, *C. oreganus*, *C. mitchelli*, *C. l. klauberi* and *C. tigris* [3,15,20,21]. The activity of SP is responsible for coagulation disorders, fibrinolysis, alterations in blood pressure, and platelet aggregation after snake bite envenomation [22,23,24], and represents an important component in the venoms of *C. atrox*, *C. tigris*, *C. molossus*, *C. culminatus*, and *C. simus* [9,14,15,25]. Along with PLA_2_, SP and SVMP are the most abundant haemotoxic enzyme families in *Crotalus* venom, representing up to half of the protein content in some species [6,14]. It was recently observed that there is a positive correlation between SVMP abundance and the hemorrhagic/proteolytic activities of the venom [21,26]. L-amino acid oxidases, C-type lectin-like proteins, and disintegrins [14,15,27] are also associated with coagulation disorders by inducing the destruction of fibrinogen or inhibiting the formation of coagulation complexes [22,28]. In addition to the venom composition and interspecies variation, intraspecies variation in the relative abundance of each protein family has previously been observed [25,26]. The differences can be explained mainly in terms of the geographic distribution of the species.

Among studied species, there are scarce reports for most Mexican endemic *Crotalus* species. This gap of information needs to be covered because of the high variability found in the venom composition among *Crotalus* and the consequences for the signs and symptoms of envenoming [29,30]. Studies that have been conducted on the venoms of Mexican species include the comparison of the biological activities in venoms of *C. aquilus* and *C. lepidus* [3], the ontogenetic changes in the venom of *C. polystictus* and *C. molossus* [9,31], and the hemotoxic effects in *C. aquilus*, *C. polystictus*, and *C. molossus* [6].

*Crotalus aquilus C. triseratus* and *C. ravus*, are closely related species and members of the *triseriatus* group [32]. Snakes of the *triseriatus* group inhabit the Mexican highlands. They are highly defensive toward humans, particularly *C. ravus* [33] which lives in the surroundings of Mexico City, one of the most populated areas in the world, and causes close to 50% of rattlesnake bites that require medical attention in this area [34,35]. *C. aquilus* and *C. triseriatus* were previously considered the same species until they were separated into sister species [36,37]. *Crotalus ravus* is placed in a more basal position in the phylogenetic tree within the *triseriatus* group, and the three taxa are far from *C. molossus*, that belongs to the *durissus* group [32]. We hypothesized that the venom of *C. triseriatus* and *C. aquilus* must share more protein features compared to *C. ravus*, and even more when compared to *C. molossus*, following existing phylogenies.

To understand the venom composition of Mexican rattlesnakes, we qualitatively and quantitatively characterized the venom proteins of three Mexican endemic species of the *triseriatus* group from the central part of the high plateau of Mexico—*C. aquilus* (Queretaran Dusky Rattlesnake, *C. triseriatus* (Mexican Dusky Rattlesnake), and *C. ravus* (Mexican Pygmy rattlesnake) [38]—and compared these with the well-known *C. molossus* (Black-tailed rattlesnake), widely distributed in Mexico and southern USA. [39]. The general composition of venoms from *C. molossus* and *C. aquilus* have been previously reported, including some biological activities and ontogenetic changes [3,6,9,40], but existing research on *C. triseriatus* and *C. ravus* has mainly focused on their geographical distribution and genetic diversity [41,42,43,44].

## 2. Results

### 2.1. Protein Profile of Venoms

**Figure 1:**
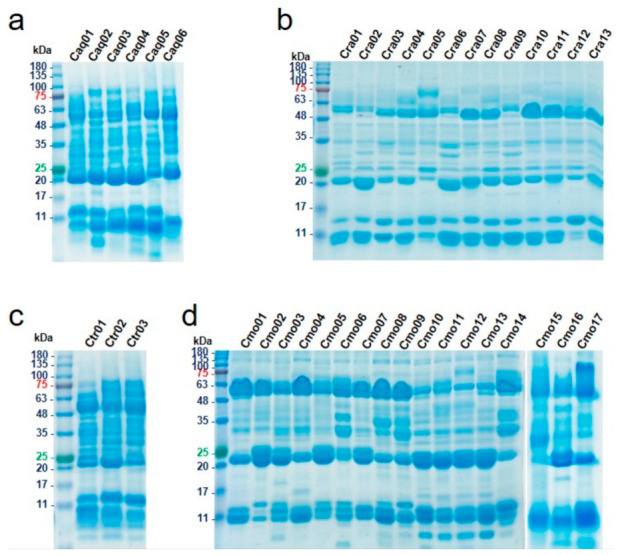
Protein profile of the venom obtained from each species of *Crotalus.* (**a**). *C. aquilus* (Caq01–Caq06), (**b**). *C. ravus* (Cra01–Cra13), (**c**). *C. triseriatus* (Ctr01–Ctr03) and (**d**). *C. molossus* (Cmo01–Cm017). The place of origin of the sampled specimen is detailed in Table A1.

The protein profile of the venom obtained from each species of *Crotalus* can be observed in Figure 1.

### 2.2. Identification of Proteins of Venoms

**Figure 2:**
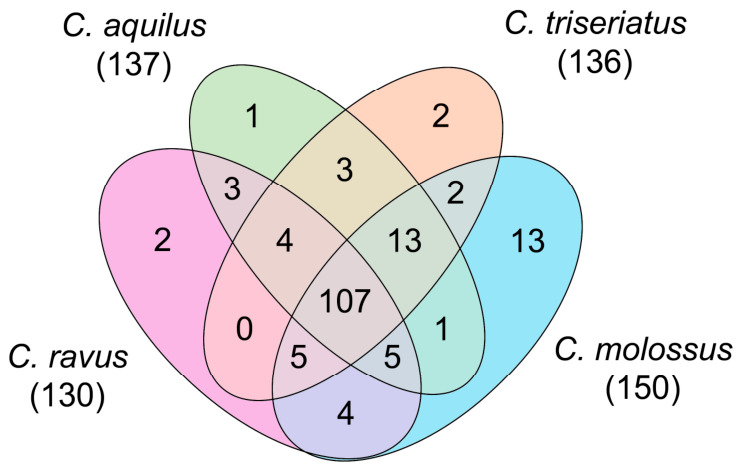
Number of unique and shared proteins identified among the four species of *Crotalus* analyzed. The total number of proteins identified in each species is indicated in parenthesis (Table S1).

Comparison of LC-MS/MS results against databases at the genus level allowed the identification of 165 proteins: 137 in *C. aquilus*, 136 in *C. triseriatus*, 130 in *C. ravus*, and 150 in *C. molossus* (Figure 2, Table S1). Proteins were classified into 19 known protein families (Table 1 and Table S1)—18 of these families were represented in all four species. Cysteine peptidase (CP) was the only family not shared by the four species; it was absent in *C. triseriatus* and *C. molossus*. The most abundant protein types were SP (38 proteins) and SVMP (28 proteins) from which 19 and 25 proteins respectively were common to all species.

Of the 165 proteins identified (Table S1), 107 proteins were shared by the four species, SVMP and SP prevailing (Figure 2 and Table S1); and 13 proteins were exclusive to *C. molossus*, including: SP, C-type lectin (CTL) and SVMP. *C. aquilus*, *C. triseriatus*, and *C. ravus* showed only four shared proteins and the sister taxa *C. aquilus* and *C. triseriatus* shared three proteins. Unexpectedly, the largest number of proteins was shared between *C. triseriatus* and *C. molossus*, not related taxa.

**Figure 3:**
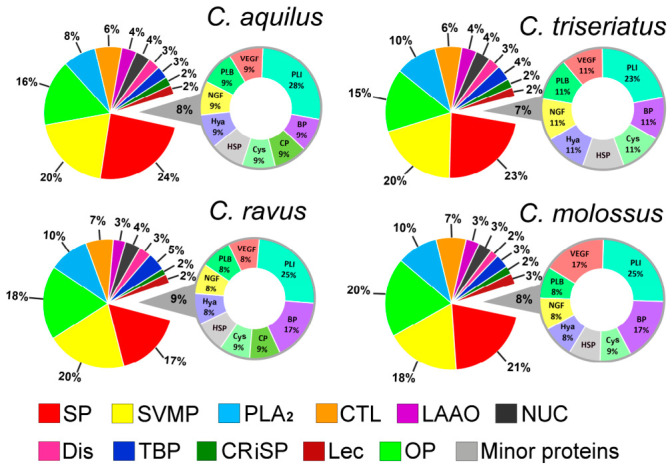
Percentage of protein families found in each *Crotalus* species by LC-MS/MS. Less common proteins are grouped as minor proteins. Abbreviations as described in Table 1.

The most common protein families were SP in *C. aquilus* and *C. triseriatus* (24%), and SVMP in *C. ravus* (22%) and *C. molossus* (20%) (Figure 3). Other relevant proteins found in all species were PLA_2_ (8–10%) and CTL (6–8%). Proteins that composed 2–4% of the content from each venom included Cysteine-Rich Secretory Protein (CRiSP), Disintegrins (Dis), L-amino Acid Oxidase (LAAO), lectins, Toxin Biosynthesis Proteins (TBPs), and Nucleic Acid-Degrading Enzymes (NUCs), also known as nucleases (Figure 3). Nerve Growth Factor (NGF), Phospholipase B (PLB), Hyaluronidase (Hya), and Cystatins are less abundant in all venoms (less than 1%). Vascular Endothelial Growth Factor (VEGF) represented 0.8% of proteins in all venoms, except in *C. molossus* that had 1.5%.

### 2.3. Correlation of Proteins Among Crotalus Species

**Figure 4:**
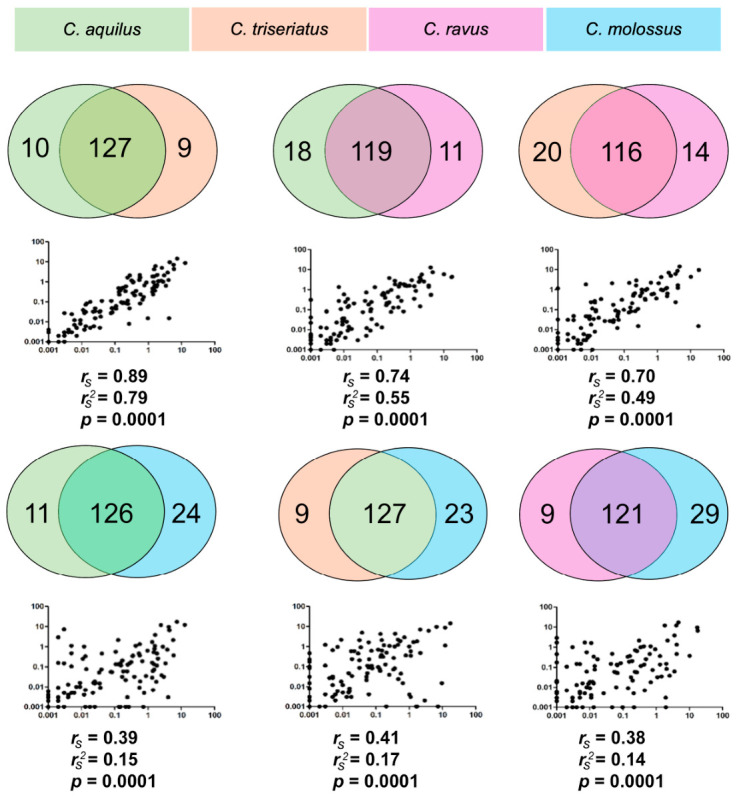
Correlation tests of paired proteins profiles identified by LF-MS/MS among *Crotalus* species. Venn diagrams show the number of shared and unique proteins for each species. The Spearman rank correlation coefficient (*r_s_*), determination coeficients (*r_s_*^2^) and *p*-value are indicated below each pair of species compared. Protein intensity, according to Label-free quantification, was represented on the logarithmic scale on the X and Y axis to illustrate descriptive trends in protein abundance across species.

According to the intensities detected by Label-free quantification (LFQ), PLA_2_ were the most abundant proteins in *C. ravus* (47.2%), followed by SVMP (29.6%); in *C. aquilus*, SVMP and SP represented half of the total proteins in the venom (28.6% and 22.8% respectively); and in *C. triseriatus* and *C. molossus*, the predominant proteins were SVMP with 45.6% and 47.5%, respectively. Serine Protease was the most prevalent protein family; however, these proteins had a wide variation in abundance among the four species, ranging from 6.5% to 22.8% (Table 1). The highest correlation of protein intensity % profiles was observed between venoms of *C. aquilus* and *C. triseriatus* (Spearman rank correlation coefficient *r_s_* = 0.89; *p* = 0.0001). In contrast, the three *triseriatus* group species showed the lowest correlation when compared to *C. molossus*, although still significant (*r_s_* = 0.38–0.41; *p* = 0.0001) (Figure 4). The graphs provide a descriptive framework to explore general patterns of interspecific similarities and differences in venom proteomes, complementing the qualitative overlap observed in Venn diagrams.

### 2.4. Proteins in Phylogeny

**Figure 5:**
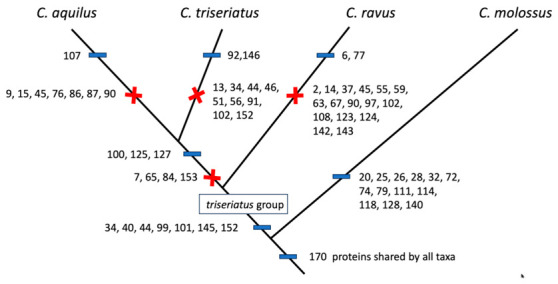
Optimization of proteins within the *triseriatus* group in a pruned tree [32] using ACCTRAN. Blue lines correspond to shared derived proteins; red crosses are reversals (protein loss). Numbers correspond to protein labels in Table S1.

According to phylogenetic analysis of the genus *Crotalus*, *C. triseriatus* and *C. aquilus* are closely related, and *C. ravus* to a lesser extent, whereas *C. molossus* is in a different clade with different ancestry [32]. Figure 5 shows several shared derived features (evolution and loss) of protein types along the tree. All four species shared 107 proteins derived from ancestry (Figure 5, Table S1). The *triseriatus* group evolved four unique proteins that supports the group on the base of venom data: Disintegrine sasaimin (40), two types of Serine endopeptidase (99, 101) and SVMP-CohPH-2 (145); it also shares Cathepsin S-like protein (34), Filamin-A isoform 9 (44) and Venom factor (152), that are later lost in *C. triseriatus*. *Crotalus aquilus* and *C. triseriatus*, as sister species, share three proteins: Serine endopeptidase (100), Serine proteinase 7 (125) and Serine proteinase 9 (127), but the clade is also defined by the loss of Alpha-enolase (7), Lectine beta-CohLL-2 (65), Phospholipase A_2_ inhibitor 31 kDa subunit-like (84), and Vespryn 1b (153).

While *C. aquilus* showed a single unique protein, a Serine endopeptidase (107), *C. triseriatus* and *C. ravus* are defined by two unique proteins each: Progesterone-induced-blocking factor 1-like (92) and Thrombin-like enzyme gyroxin B1.4 (146) for *C. triseriatus*, and Alpha globin (6) and Peptidyl-prolyl cis-trans isomerase (77) for *C. ravus*. All three species are characterized by the loss of many proteins at the origin of each lineage. *Crotalus aquilus* lost Aminopeptidase (9), Basic phospholipase A_2_ (15), Ganglioside GM2 activator (45), Nuclear receptor coactivator 7 (76), Phospholipase A_2__1 (86), Phospholipase A_2__1 (87), and Plasma protease C1 inhibitor-like protein (90). *Crotalus triseriatus* lost Annexin (13), Cathepsin S-like protein (34), Filamin-A isoform 9 (44), Glia-derived nexin-like protein (46), Hemopexin (51), Kallikrein-CohID-4 (56), Plastin-3-like protein (91), and Venom factor (152). Finally, *C. ravus* lost the most proteins among the *triseriatus* group taxa: Acidic phospholipase A_2_ (2), ATP synthase subunit beta (14), ATP synthase subunit beta (14), CRiSP (37), Ganglioside GM2 activator (45), Inactive snake venom serine proteinase 13 (55), LAAO Cdc18 (59), Lec alpha-CohCI-4 (63), Metalloproteinase (67), Plasma protease C1 inhibitor-like protein (90), Secretory phospholipase A_2_ (97), four forms of Serine endopeptidase (102, 108, 123, 124), Snake venom serine proteinase (142), and Snake venom serine proteinase 9 (143) (Figure 5, Table S1).

There are 13 derived characters unique to *C. molossus* (Figure 2, Table S1); however, these proteins may be shared with some other snakes not included in our analysis.

## 3. Discussion

The comparative proteomic analysis of venoms from four *Crotalus* species revealed differences both at the qualitative and quantitative levels. *Crotalus molossus* is the species with the most complex venom composition, and *C. ravus* with the least, while venoms of *C. aquilus* and *C. triseriatus* are similar. It is noticeable that 28 of the 111 proteins shared by *C. aquilus*, *C. ravus* and *C. triseriatus*, match with *C. adamanteus* and 30 with *C. horridus*, even though these species are phylogenetically distant from the four Mexican species analyzed in this work, suggesting that these venom proteins are primitive among the venom composition of rattlesnakes.

When contrasting the qualitative and quantitative analysis of venom components, SP represented the major toxin family in *C. aquilus* and *C. triseriatus*, similarly to what has been reported for *C. tigris* [15]. Label-free quantification revealed substantial quantitative differences between species, with SPs accounting for ~23% of venom composition in *C. aquilus*, compared with ~11% in *C. triseriatus*, and ~6% in *C. ravus* and *C. molossus* (Figure 3 and Table 1). High levels of SVMP and lower SP were reported in *C. molossus* because of ontogenetic changes [9]. Similarly, the venoms of *C. basiliscus* and *C. atrox* are also abundant in SVMP, which are responsible for their hemorrhagic activity [14,45].

*Ctotalus ravus* venom showed a remarkable predominance of PLA_2_ (47.2% of its venom composition, Table 1), albeit only 10% of the total number of proteins belongs to this family (Figure 3), this profile is similar to that reported for *C. durissus* [46]. PLA_2_ represented a much smaller proportion of the venom of the other three Mexican species analyzed (13–21%). The neurotoxic effects of *C. scutulatus* venom attributed to PLA_2_ are poorly or not neutralized by a commercial antivenom [5]. As neurotoxins in snake venom are composed of a basic subunit with phospholipase activity and an acidic subunit without enzymatic properties, further analyses are required to discriminate the proportion of non-toxic phospholipases A_2_ and the PLA_2_ corresponding to the basic subunits of neurotoxins [47]. In this context, there is a need to characterize the toxin components as well as the minor components to fill the gaps in the knowledge in the field of toxicology and pharmaceutical interests. Grabowsky et al. [7] reported that the main differences in the montane rattlesnake venoms are largely due to the differential expression of the amounts and subtypes of common toxin families, rather than to the presence of new toxin families. The evolutionary dynamics of neurotoxin gain, retention, and loss remain a key research frontier with implications for venom biology, evolutionary theory, and the design of improved therapeutic interventions [47].

The venom composition of *C. triseriatus* and *C. ravus* was characterized here for the first time. The unique properties of the molecules found in these venoms make them valuable resources for drug development, as well as useful immunogens to produce specific antivenoms or for various potential physiological applications. Venomous animals have long been identified as a rich source of bioactive compounds with various biological activities. Several prominent drugs derived from animal venoms have been approved by the US Food and Drug Administration (FDA) for human use, with others either undergoing or progressing through clinical trials [48]. Antibacterial and hemolytic activity have been described in the venoms of these species [43], and the observed variation in hemolytic activity could be attributable to the different proportions of SVMP present [44]. Differences between the hemolytic activity observed could be attributable to the unequal proportion of SVMP found. LAAO activity in snake venoms has been associated with hemorrhagic effects, induction of apoptosis, and platelet aggregation [28]. LAAO represented only 3–4% of the proteins in the four venoms analyzed compared to the 19% of the total protein in *C. aquilus* venom (Table 1), in which it represents the third most abundant protein in this species.

C-type lectins inhibit or activate platelets by binding to receptors with pro/anti-coagulant and pro/anti-thrombotic activities [49,50]. These proteins represented 6–7% of protein content in the venoms studied here (Figure 3). These levels were higher than those reported for *C. atrox* [14], *C. durissus* [46], *C. viridis* [51], and *C. simus* [25]. Abundance of CRiSP in *C. aquilus*, *C. triseriatus*, and *C. molossus* was higher than the mean reported for other *Crotalus* species [30], but lower in the venoms of *C. atrox* [14] and *C. viridis* [51]. CRiSP is widely distributed in snake venom and contributes to blocking smooth muscle contraction in prey [52]. In contrast to other *Crotalus* species, bradykinin-potentiating peptides were not detected in any of the four venoms studied [53,54]. Angiotensin-Converting Enzyme (ACE) was present all species (Table 1), suggesting a role in the increase in blood pressure as seen in other venomous organisms [55,56], but its specific biological role in snake venoms remains unclear and warrants further investigation [55]. Myotoxin was found only in *C. molossus*. Since the myonecrosis induced by myotoxins is different from that produced by the cardiotoxins and PLA_2_ [57], myotoxins should be considered an important target to prevent disabilities after snakebites. Also, a venom factor-like protein was detected in *C. aquilus* and *C. ravus*. Proteins annotated as venom factors are homologous to cobra venom factor (CVF), a complement-modulating protein originally described in elapid venoms that can activate and deplete complement components, contributing to immune modulation during envenomation [58,59]. However, the biological relevance and functional activity of venom factor-like proteins in *Crotalus* venoms remain unknown and require experimental validation.

The close relationship of *C. triseriatus*, *C. aquilus* and *C. ravus* in their venom composition [41] agrees with the inclusion of the three taxa in the *triseriatus* species group (Figure 5). The high similarity found between *C. aquilus* and *C. triseriatus* correlates with their close phylogenetic relation, proposed as a sister relationship between the species [41]. It seems to be clear that the evolution of the venom composition is more related to the loss of previously existing proteins in terminal taxa than to the evolution of novel proteins associated with the emergence of new species (Figure 5), a pattern here called the *terminal taxa protein loss phenomenon*. This pattern has been reported previously in a broader sense when studying DNA venom coding for specific venom proteins [60,61]. Venom protein components originated first in lizards, and then in snakes, which developed complex venom systems [62]. Surprisingly, within venomous snake evolution, only a few new molecules originated, and at the species level, venom cocktails evolved without several ancestral venom components [60]. Most proteins that structure the evolution of venom in the *triseriatus* group are minor proteins and the unexpected great similarities found between *C. triseratus* and *C. molossus* venoms are shared primitive proteins, lost in *C. aquilus* and *C. ravus*. Venom composition differences within the *triseriatus* group could be related to the geographical barriers among taxa that may lead to changes in behavior and diet, and genetic breaks [63,64]. We emphasize the necessity for future research employing biological replicates considering geography, sex and ontogeny to determine the composition of venoms at a regional level to produce biological agents that will efficiently neutralize their toxic effects, preventing fatalities and long-term consequences.

Despite the broad biomedical applications of snake venoms (anticoagulant, cytotoxic, anti-inflammatory, antiviral, procoagulant, hemostatic, antinociceptive, cytolytic, thrombolytic, antibacterial, and immunogenic) their compositional variability complicates the standardization of products for clinical use. Looking ahead, advancements in biotechnology and the use of proteomics, transcriptomics, metabolomics will likely drive further innovation in this field. These technologies will enable more precise identification of venom components and their interactions with biological systems [65].

## 4. Conclusions

To our knowledge, this work represents the first report comparing venoms at the quantitative level among Mexican crotalids. Up to 165 proteins were identified in the venoms of *C. aquilus*, *C. triseriatus*, *C. ravus*, and *C. molossus*. Venom composition seems to be similar among all *Crotalus* species, but LFQ-MS/MS analysis revealed differences between venoms, which may be responsible for the different envenomation symptoms and patient treatment response. Knowledge of the venom composition of endemic species will contribute to improve envenomation treatment. We hope our findings will stimulate antivenom manufacturers to include venoms from endemic species in the immunizing mixture to produce better antivenoms according to specific regional needs, and researchers to study venoms in a phylogenetic context to understand the distribution of venoms between taxa based on ancestry and not only in overall similarity. These can facilitate the elaboration of new antivenoms upon shared derived proteins.

## 5. Materials and Methods

### 5.1. Snakes and Venom

Venom samples of *C. ravus. C. triseriatus*, *C. aquilus*, and *C. molossus* were obtained from adult specimens previously collected at Ciudad de México and the Mexican states of Estado de México, Hidalgo, San Luis Potosí, Guanajuato, and Zacatecas (Table A1). Snakes were maintained in captivity in herpetariums with controlled environmental conditions at Escuela Nacional de Ciencias Biológicas at Instituto Politécnico Nacional, and Facultad de Ciencias and Facultad de Estudios Superiores (FES) Iztacala at Universidad Nacional Autónoma de México. The rattlesnakes were carefully identified by experts and following specialized identification keys [66]. Venom was extracted manually from snakes in excellent health, using sterile plastic containers. Venom samples were centrifuged at 12,000 rpm for 15 min at 4 °C to eliminate cellular debris, and stored at −70 °C.

For Mass Spectrometry (MS), 10 µg of venom from each of several specimens were pooled to conform a species sample, and 20 µg from the resulting pool was submitted for mass spectrometry analysis. The study included six specimens of *C. aquilus*, 13 specimens of *C. ravus*, three specimens of *C. triseriatus*, and 17 specimens of *C. molossus*. Although the pooling strategy masked intraspecific diversity (geographic, sexual and ontogenetic), it was employed to minimize individual variability and to obtain a species-level representation of venom composition.

### 5.2. SDS-PAGE

Electrophoresis was used to compare the venom obtained from the different species. For SDS-PAGE, 20 μg of each crude venom was loaded in 13% polyacrylamide gels. Gels were stained with Coomassie brilliant blue R-250.

### 5.3. In-Gel Trypsin Digestion

Proteomic analysis was performed by an external service provider (Creative Proteomics, Shirley, NY, USA). SDS-PAGE lanes containing 20 μg of each venom sample were cut into 1 mm^3^ cubes and subjected to reduction with 10 mM DTT (Sigma-Aldrich, Inc., St. Louis, MO, USA; Cat. D9779), alkylation with 50 mM Iodoacetamide (Sigma-Aldrich, Inc., St. Louis, MO, USA; Cat. I6125) and trypsin digestion (Promega, Madison, WI, USA; Cat. V5280) before LC-MS/MS analysis. Gel slices were treated with 50 mM ammonium bicarbonate/acetonitrile solution (1:2, *v*/*v*). Peptides were finally resuspended in 20 μL of 0.1% formic acid.

### 5.4. Nanoscale Liquid Chromatography Coupled to Tandem Mass Spectrometry

The resulting tryptic peptides were analyzed by nanoscale liquid chromatography coupled to tandem mass spectrometry (nano LC-MS/MS) using an Ultimate 3000 nano UHPLC system (Thermo Fisher Scientific, Waltham, MA, USA) coupled with a Q Exactive HF mass spectrometer (Thermo Fisher Scientific, Waltham, MA, USA), with a trapping column PepMap C18 100Å (100 μm × 2 cm, 5 μm) (Thermo Fisher Scientific, Germering, Germany; Cat. No. 164199) and an analytical column PepMap C18 100Å (75 μm × 50 cm, 2 μm) (Thermo Fisher Scientific, Germering, Germany; Cat. No. 164942). The flow rate was 250 nL/min. Buffer A: 0.1% formic acid in water; and buffer B: 0.1% formic acid in 80% acetonitrile were applied as follows: a linear gradient from 2 to 8% buffer B in 3 min, from 8% to 20% buffer B in 50 min, from 20% to 40% buffer B in 26 min, then from 40% to 90% buffer B in 4 min. The full scan was performed between 300 and 1650 *m*/*z* at the resolution 60,000 at 200 *m*/*z*, the Automatic Gain Control (AGC) target for the full scan was set to 3 × 10^6^.

The MS/MS scan was operated in the Top 20 mode using the following settings: resolution 15,000 at 200 *m*/*z*; A.G.C. target 1 × 10^5^; maximum injection time 19 ms; normalized collision energy at 28%; isolation window of 1.4 Th; and dynamic exclusion 30 s. Label-Free based Quantitative Proteomics (LFQ-MS/MS) is an effective method to determine and compare the relative amount of protein in various biological samples since quantification is determined by either spectral counting or chromatographic peak intensity measurement. This technology has been successfully applied before comparing protein composition in complex biological samples [67,68,69].

The use of a single LC–MS/MS technical run per species precluded the assessment of technical reproducibility and the calculation of variance metrics. Furthermore, specific LFQ normalization settings remain undefined due to the use of an external service.

### 5.5. Data Analysis

Raw MS files were analyzed and searched against the *Crotalus* protein database using Maxquant (version 1.5.6.5) and the Andromeda search engine V. 1.6.2.6 (Max Planck Institute of Biochemistry, Martinsried, Germany, 2016), extracted from UniProt Knowledgebase (UniProtKB; released 2018-12) as previously validated [70]. Results obtained were filtered accepting only proteins with at least one unique peptide and False Discovery Rate (FDR) ≤ 1%. The parameters were set as follows: the protein modifications were Carbamidomethylation (C) (fixed), oxidation (M) (variable); the enzyme specificity was set to trypsin with up to two missed cleavages allowed; the precursor ion mass tolerance was set to 10 ppm, and MS/MS tolerance was 0.5 Da. FDR was controlled at 1% at both peptide and protein levels using default MaxQuant settings.

Label-free quantification (LFQ) was carried out using the MaxQuant LFQ algorithm, which incorporates internal normalization procedures. Detailed parameters of the normalization algorithm are not available, as the analysis was conducted by an external service provider.

### 5.6. Statistical Analysis

Spearman rank correlation coefficients (*r_s_*) and coefficients of determination (*r_s_*^2^) were calculated to assess the degree of association between protein intensities obtained by LFQ in pairwise comparisons among species. Raw protein intensities (Table S1) were analyzed using the library(Hmisc) [71] in R Core Team 2025 [71]. Statistical significance was assessed using two-tailed tests, with *p* < 0.05 considered significant. The *p* values corresponding to each correlation are reported alongside the *r_s_* values, and *r_s_*^2^ is interpreted as the proportion of variance shared between datasets. Correlation graphs provide a visual approach to correlations for which data were log-transformed and compared using GraphPad Prism 8 for Windows.

For the phylogenetic character analyses, we translated Table S1 into an incidence matrix and optimized character distribution using the Trace Character History option in Mezquite 4.03 [71], preferring the acceleration transformation optimization (ACCTRAN) for ambiguous characters, as this favors the loss of a protein—more plausible in protein evolution—over its convergent evolution among two clades. The analysis was done using a pruned tree from Myers et al. [32] and drawn by hand.

## Figures and Tables

**Table 1:** Number of proteins and relative protein abundance (%RPA) of venom protein families identified in each *Crotalus* species.

Protein Family	Total Identified Proteins	Shared Proteins ^Δ^	*C. aquilus*		*C. triseriatus*		*C. ravus*		*C. molossus*	
			n	% RPA ^‡^	n	% RPA ^‡^	n	% RPA ^‡^	n	% RPA ^‡^
BP	2	1	1	0.03728%	1	0.01787%	2	0.01103%	2	0.20497%
CP	1	0	1	0.00192%	0	0.00000%	1	0.00034%	0	0.00000%
CRISP	3	2	3	6.47016%	3	7.71466%	2	2.39910%	3	3.24449%
CTL	12	9	9	2.26579%	9	3.78854%	9	2.35635%	11	1.81937%
CYS	1	1	1	0.00302%	1	0.00310%	1	0.00580%	1	0.00121%
DIS	4	3	4	0.50740%	4	0.75160%	4	1.96566%	3	1.34946%
HSP	1	1	1	0.00035%	1	0.00128%	1	0.00348%	1	0.00248%
HYA	1	1	1	0.10747%	1	0.06123%	1	0.06537%	1	0.13387%
LAAO	5	4	5	19.05046%	5	14.00283%	4	6.18332%	5	12.58166%
LEC	4	2	3	0.05641%	3	0.17357%	3	0.06852%	4	0.93967%
NGF	1	1	1	0.12981%	1	0.30760%	1	0.43405%	1	0.13746%
NUCs	5	5	5	0.56762%	5	0.84804%	5	0.73129%	5	3.26596%
PLA_2_	15	9	11	16.02219%	14	13.02339%	13	47.28635%	15	21.19788%
PLB	1	1	1	1.98703%	1	1.07517%	1	0.48873%	1	0.25618%
PLI	3	2	3	0.03773%	2	0.00507%	3	0.00070%	3	0.02504%
SPs	38	19	33	22.85323%	31	11.42513%	22	6.65675%	31	6.74821%
SVMP	28	25	27	28.65264%	27	45.68686%	26	29.62369%	27	47.52601%
TBPs	6	4	4	0.28651%	5	0.11856%	6	0.12268%	5	0.16466%
VEGF	2	1	1	0.87448%	1	0.92119%	1	1.44373%	2	0.04232%
OPs	32	16	22	0.08849%	21	0.07430%	24	0.15307%	29	0.55457%
Total	165	107	137	100%	136	100%	130	100%	150	100%

BP: Blood Protein; CP: Cystein Peptidase; CRiSP: Cysteine-Rich Secretory Protein; CTL: C-Type Lectin; Cys: Cystatine; Dis: Disintegrin; HSP: Heat Shock Protein; Hya: Hyaluronidase; LAAO: L-Amino Acid Oxidase; Lec: Lectin; NGF: Nerve Growth Factor; NUCs: Nucleic Acid-Degrading Enzymes; PLA_2_: Phospholipases A_2_; PLB: Phospholipase B; PLI: Phospholipase Inhibitor; SPs: Serin Proteases; SVMP: Snake Venom Metalloprotease; TBPs: Toxin Biosynthesis Proteins; VEGF: Vascular Endothelial Growth Factor; OPs: Other Proteins. ^Δ^ Shared proteins among the four species. ^‡^ % RPA for each family was calculated as the sum of % intensity of each protein based on Label-free quantification.

## Data Availability

The original contributions presented in this study are included in the article/supplementary material. Further inquiries can be directed to the corresponding authors.

## Supplementary Materials

The following supporting information can be downloaded at: https://www.mdpi.com/article/10.3390/toxins18060256/s1, Table S1: Identified proteins *in Crotauls aquilus*, *C. triseriatus*, *C. ravus* and *C. molossus*. Abbreviations: **Taxon**: *C.*: *Crotalus*; *Ce.*: *Cerrhophideon*, *S.: Sistrurus.* **Protein family**: BP: Blood Protein; CP: Cystein Peptidase; CRiSP: Cysteine-Rich Secretory Protein; CTL: C-Type Lectin; Cys: Cystatine; Dis: Disintegrin; HSP: Heat Shock Protein; Hya: Hyaluronidase; LAAO: L-amino Acid Oxidase; Lec: Lectin; NGF: Nerve Growth Factor; NUCs: Nucleic Acid-Degrading Enzymes; PLA_2_: Phospholipases A_2_; PLB: Phospholipase B; PLI: Phospholipase Inhibitor; SPs: Serin Proteases; SVMP: Snake Venom Metalloprotease; TBPs; Toxin Biosynthesis Proteins; VEGF: Vascular Endothelial Growth Factor; OPs: Other Proteins. *Key shared proteins*: A: *C. aquilus*; T: *C. triseriatus*; R: *C. ravus*; M: *C. molossus*.

## Abbreviations

The following abbreviations are used in this manuscript:

ACE	Angiotensin-Converting Enzyme
BP	Blood Protein
CP	Cysteine Peptidase
CRiSP	Cysteine-Rich Secretory Protein
CTL	C-type lectin
Cys	Cystatine
Dis	Disintegrin
FDR	False Discovery Rate
HSP	Heat Shock Protein
Hya	Hyaluronidase
LAAO	L-amino Acid Oxidase
Lec	Lectin
LFQ	Label-free quantification
LC-MS/MS	Liquid Chromatography Coupled to tandem Mass Spectrometry
NGF	Nerve Growth Factor
NUCs	Nucleic Acid-Degrading Enzymes
OPs	Other proteins
PLA_2_	Phospholipases A_2_
PLB	Phospholipase B
PLI	Phospholipase Inhibitor
SEMARNAT	Secretaría del Medio Ambiente y Recursos Naturales
SPs	Serine Proteases
SVMP	Snake Venom Metalloprotease
TBPs	Toxin Biosynthesis Proteins
VEGF	Vascular Endothelial Growth Factor
WHO	World Health Organization

## Appendix A

**Table A1:** Place of origin of the specimens sampled.

	Specimen	Locality
*C. aquilus*	Caq01	Huichapan, Hidalgo
	Caq02	San Luis Potosí
	Caq03	Ecatepec, EdoMex
	Caq04	Querétaro
	Caq05	San Luis de la Paz, Guanajuato
	Caq06	San Luis de la Paz, Guanajuato
*C. ravus*	Cra01	Texcoco, EdoMex
	Cra02	Texcoco, EdoMex
	Cra03	Xochimilco, CDMX
	Cra04	Oaxaca
	Cra05	Valle de México
	Cra06	Milpa Alta, CDMX
	Cra07	Milpa Alta, CDMX
	Cra08	Milpa Alta, CDMX
	Cra09	Milpa Alta, CDMX
	Cra10	Milpa Alta, CDMX
	Cra11	Milpa Alta, CDMX
	Cra12	Milpa Alta, CDMX
	Cra13	Milpa Alta, CDMX
*C. triseriatus*	Ctr01	Ajusco, CDMX
	Ctr02	Sierra de Guadalupe, Edo Mex
	Ctr03	Sierra de Guadalupe, Edo Mex
*C. molossus*	Cmo01	Ajacuba, Hidalgo
	Cmo02	Huichapan, Hidalgo
	Cmo03	Chapa de Mota, EdoMex
	Cmo04	Chapa de Mota, EdoMex
	Cmo05	Mazapil, Zacatecas
	Cmo06	Mazapil, Zacatecas
	Cmo07	Zacatecas, Zacatecas
	Cmo08	Mazapil, Zacatecas
	Cmo09	Oaxaca
	Cmo10	Pedregal de San Ángel, CDMX
	Cmo11	Pedregal de San Ángel, CDMX
	Cmo12	Pedregal de San Ángel, CDMX
	Cmo13	Pedregal de San Ángel, CDMX
	Cmo14	Pedregal de San Ángel, CDMX
	Cmo15	Jardín botánico-UNAM
	Cmo16	Jardín botánico-UNAM
	Cmo17	Jardín botánico-UNAM
