# Supplementary material for: Qualitative and Quantitative Proteomic Analysis of Venoms from Mexican Rattlesnakes
Source: Toxins (Basel). 2026 Jun 5;18(6):256. doi: 10.3390/toxins18060256 (PMC13308136; doi:10.3390/toxins18060256)
Supplement: Supplementary file 1 [file toxins-18-00256-s001.zip › toxins-4265962-supplementary.pdf]

Qualitative and Quantitative Proteomic Analysis of Venoms from Mexican Rattlesnakes

Lizbeth Hernández-Ancheyta, Víctor Hugo Reynoso, Juan Carlos López-Vidal, Javier Hernández-Sánchez, Karen Delgadillo-Gutiérrez, María Lilia Domínguez-López and Julieta Luna-Herrera

Table S1. Identified proteins in *Crotauls aquilus, C. triseriatus, C. ravus* and *C. molossus.* Abbreviations: **Taxon**: *C.*: *Crotalus*; Ce.: *Cerrhophideon*, *S.*: *Sistrurus*. **Protein family**: BP: Blood Protein; CP: Cystein Peptidase; CRiSP: Cysteine-Rich Secretory Protein; CTL: C-Type Lectin; Cys: Cystatine; Dis: Disintegrin; HSP: Heat Shock Protein; Hya: Hyaluronidase; LAAO: L-amino Acid Oxidase; Lec: Lectin; NGF: Nerve Growth Factor; NUCs: Nucleic Acid-Degrading Enzymes; PLA$_2$: Phospholipase A$_2$; PLB: Phospholipase B; PLI: Phospholipase Inhibitor; SPs: Serin Proteases; SVMP: Snake Venom Metalloprotease; TBPs; Toxin Biosynthesis Proteins; VEGF: Vascular Endothelial Growth Factor; OPs: Other Proteins. **Key shared proteins**: A: *C. aquilus*; T: *C. triseriatus*; R: *C. ravus*; M: *C. molossus*.

No.	Protein ID	Protein name	Taxon	Protein family	Sequence coverage [%]	Mol. weight [kDa]	*Crotalus aquilus*		*Crotalus triseriatus*		*Crotalus ravus*		*Crotalus molossus*		Key shared Proteins
							% of intensity	Sequence coverage [%]	% of intensity	Sequence coverage [%]	% of intensity	Sequence coverage [%]	% of intensity	Sequence coverage [%]	
1	T1DBJ9	78 kDa glucose-regulated protein	*C. horridus*	OP	7.5	72.104	0.00100%	6	0.00272%	7.5	0.00169%	3.2	0.00096%	4.3	ATRM
2	Q800C3	Acidic phospholipase A2	*C. viridis*	PLA$_2$	29	15.642	0.00030%	18.1	0.00058%	23.9	0.00000%	13	0.20401%	29	ATM
3	Q7ZTA7	Acidic phospholipase A2 CoaPLA2	*C. oreganus*	PLA$_2$	39.9	15.549	0.08342%	23.2	0.03034%	23.2	0.00261%	30.4	0.00008%	23.2	ATRM
4	T1E7F6	Actin, alpha skeletal muscle	*C. horridus*	OP	31.3	42.051	0.00122%	28.6	0.00350%	25.7	0.00102%	23.6	0.00616%	28.6	ATRM
5	T1DP26	Actin, cytoplasmic 1	*C. horridus*	OP	46.9	41.736	0.03400%	37.3	0.03073%	28.8	0.06840%	40.5	0.05152%	46.9	ATRM
6	J3SDZ6	Alpha globin	*C. adamanteus*	BP	18.3	16.014	0.00000%	0	0.00000%	0	0.00079%	18.3	0.00000%	0	R
7	T1DDS0	Alpha-enolase	*C. horridus*	OP	8.5	47.491	0.00000%	0	0.00000%	0	0.00090%	8.5	0.00032%	8.5	RM
8	A0A0F7ZD91	Aminopeptidase	*C. adamanteus*	TBP	24	109.9	0.01212%	14.5	0.02981%	21.9	0.04783%	15.3	0.00016%	0.9	ATRM
9	T1DNX8	Aminopeptidase	*C. horridus*	TBP	7.6	110.77	0.00000%	0	0.00270%	6.7	0.00062%	1	0.01836%	5.9	TRM
10	T1E6L9	Aminopeptidase	*C. horridus*	TBP	8.2	107.4	0.00741%	8.2	0.00231%	3.9	0.00024%	0.9	0.00031%	1.7	ATRM
11	J3SE00	Angiotensin-converting enzyme	*C. adamanteus*	OP	13	148.34	0.00024%	1.5	0.00405%	5.5	0.00844%	6.4	0.06090%	12.1	ATRM
12	J3SBT6	Annexin	*C. adamanteus*	PLI	16.1	38.623	0.00233%	16.1	0.00006%	3.5	0.00012%	2	0.00914%	8.8	ATRM
13	T1DDN7	Annexin	*C. horridus*	PLI	5.9	38.523	0.00022%	3.2	0.00000%	0	0.00031%	3.2	0.00100%	5.9	ARM
14	T1E780	ATP synthase subunit beta	*C. horridus*	OP	4.5	56.561	0.00087%	2.6	0.00077%	2.6	0.00000%	0	0.00076%	4.5	ATM
15	P0CAS7	Basic phospholipase A2	*C. durissus*	PLA$_2$	30.6	14.368	0.00000%	13.2	0.00047%	14.9	2.03651%	30.6	0.08256%	14.9	TRM
16	Q8UVZ7	Basic phospholipase A2 homolog Cax-K49	*C. atrox*	PLA$_2$	60.6	15.597	0.27663%	57.7	0.00011%	30.7	0.52802%	57.7	0.00045%	47.4	ATRM
17	P21963	C-type lectin	*C. atrox*	CTL	58.5	16.291	1.48931%	58.5	1.88062%	51.1	0.00007%	6.7	0.53529%	41.5	ATRM

18	G9DCH7	C-type lectin 1	*C. oreganus*	CTL	39.4	16.395	0.00924%	11.3	0.01206%	16.9	0.06220%	12	0.00969%	34.5	ATRM
19	J3SDU9	C-type lectin 11	*C. adamanteus*	CTL	28.5	18.261	0.00266%	17.1	0.02684%	28.5	0.02606%	17.1	0.00504%	5.1	ATRM
20	J3SDV5	C-type lectin 13b	*C. adamanteus*	CTL	12.1	17.219	0.00000%	0	0.00000%	0	0.00000%	0	0.02612%	12.1	M
21	T1DH34	C-type lectin 19a	*C. horridus*	CTL	32.5	17.911	0.01416%	8.9	0.08199%	8.9	0.01443%	21	0.07670%	25.5	ATRM
22	A0A0K8RZ50	C-type lectin 2	*C. horridus*	CTL	42	17.459	0.35250%	26	0.99299%	42	1.29573%	42	0.36449%	36	ATRM
23	F8S0Y6	C-type lectin 2	*C. adamanteus*	CTL	44.5	16.836	0.13470%	39.7	0.22814%	17.8	0.01306%	22.6	0.00097%	39.7	ATRM
24	G9DCH8	C-type lectin 2	*C. oreganus*	CTL	31.8	16.788	0.08107%	31.8	0.00039%	11.9	0.61835%	31.8	0.00004%	11.3	ATRM
25	G9DCI0	C-type lectin 4	*C. oreganus*	CTL	12.1	17.295	0.00000%	0	0.00000%	0	0.00000%	0	0.08714%	12.1	M
26	J3SBN9	C-type lectin 6	*C. adamanteus*	CTL	35	16.433	0.00000%	15	0.00000%	15.7	0.00000%	6.4	0.17294%	25.7	M
27	A0A1W7RBC3	Cadam10_CTL-11	*C. adamanteus*	CTL	20.9	17.442	0.16919%	8.5	0.49330%	8.5	0.18839%	8.5	0.20697%	20.9	ATRM
28	A0A1W7RB98	Cadam10_MYO-2	*C. adamanteus*	OP	23.8	7.1664	0.00000%	0	0.00000%	0	0.00000%	0	0.02768%	23.8	M
29	A0A1W7RB89	Cadam10_SVMPIII-5	*C. adamanteus*	SVMP	24.6	67.721	0.02526%	17.3	0.03398%	11.5	0.01205%	11.5	0.01850%	9.6	ATRM
30	A0A1W7RB97	Cadam10_SVMPIII-6	*C. adamanteus*	SVMP	31.2	69.019	1.49064%	24.8	4.19182%	27.6	1.30165%	13.4	0.13641%	17	ATRM
31	A0A1W7RB66	Cadam10_SVSP-12	*C. adamanteus*	SP	37.2	28.326	1.56719%	36.8	0.35971%	33.7	0.92815%	25.6	0.79190%	24.4	ATRM
32	A0A1W7RB67	Cadam10_VEGF-1	*C. adamanteus*	VEGF	21.7	12.272	0.00000%	0	0.00000%	0	0.00000%	0	0.00740%	21.7	M
33	J3RYP4	Carboxypeptidase E-like	*C. adamanteus*	NUC	21.7	53.839	0.00609%	16.7	0.01173%	21.7	0.00511%	10	0.00467%	5.8	ATRM
34	T1DNJ9	Cathepsin S-like protein	*C. horridus*	CP	5.7	37.705	0.00192%	5.7	0.00000%	0	0.00034%	3.3	0.00000%	0	AR
35	J3S8G4	CREG1-like protein	*C. adamanteus*	OP	19.9	20.778	0.00296%	10.8	0.00230%	10.8	0.00049%	15.6	0.00056%	10.8	ATRM
36	J3RYX9	Cystatin-1	*C. adamanteus*	CYS	30.2	15.623	0.00302%	24.5	0.00310%	30.2	0.00580%	15.1	0.00121%	9.4	ATRM
37	F8S0Y4	Cysteine-rich secretory protein	*C. adamanteus*	CRISP	24.2	26.629	0.00543%	14.2	0.00268%	14.2	0.00000%	10.4	1.10168%	24.2	ATM
38	G9DCH4	Cysteine-rich secretory protein 1	*C. oreganus*	CRISP	35.7	26.55	0.76344%	22.7	0.65667%	22.7	0.20906%	22.7	0.00492%	34.5	ATRM
39	F2Q6E8	Cysteine-rich secretory protein Ch-CRPIa	*C. horridus*	CRISP	48.9	24.745	5.70129%	34.8	7.05531%	29	2.19004%	29	2.13790%	48.9	ATRM
40	C0HJM5	Disintegrin sasaimin	*Ce. sasai*	DIS	50	7.6145	0.00566%	50	0.00690%	50	0.00120%	47.2	0.00000%	23.6	ATR
41	T1DJT5	Ectonucleotide pyrophosphatase/phosphodiesterase family member 3-like protein	*C. horridus*	NUC	55.5	93.753	0.12740%	43.5	0.48199%	52.4	0.30257%	23.1	2.14957%	30.4	ATRM
42	T1DH21	Ectonucleotide pyrophosphatase/phosphodiesterase family member 6-like protein	*C. horridus*	NUC	14.6	50.364	0.00355%	9.4	0.00429%	14.6	0.00076%	1.8	0.00252%	1.8	ATRM
43	T1DN73	Elongation factor 1-alpha	*C. horridus*	OP	11.3	50.2	0.00175%	5	0.00031%	2.6	0.00257%	11.3	0.00301%	11.3	ATRM
44	A0A0K8RV79	Filamin-A isoform 9	*C. horridus*	OP	1.9	274.77	0.00122%	0.4	0.00000%	0	0.00100%	1.5	0.00000%	0	AR
45	T1E5P6	Ganglioside GM2 activator	*C. horridus*	OP	10.1	22.471	0.00000%	0	0.00199%	10.1	0.00000%	0	0.00034%	3.8	TM
46	T1DM74	Glia-derived nexin-like protein	*C. horridus*	OP	6.1	44.172	0.00050%	3	0.00000%	0	0.00017%	3	0.00390%	6.1	ARM
47	T1DH29	Glutaminyl-peptide cyclotransferase	*C. horridus*	TBP	58.2	42.369	0.24382%	58.2	0.07812%	48.4	0.06740%	38.9	0.12946%	28.3	ATRM

48	T1E6G6	Glyceraldehyde-3-phosphate dehydrogenase	*C. horridus*	OP	10.8	35.821	0.00072%	10.8	0.00014%	4.5	0.00121%	10.8	0.00182%	10.8	ATRM
49	T1E691	Heat shock protein 90a	*C. horridus*	HSP	3.6	84.127	0.00035%	1.6	0.00128%	3.6	0.00348%	3.6	0.00248%	3.6	ATRM
50	T1E6B5	Hemoglobin subunit rho	*C. horridus*	BP	12.9	16.43	0.03728%	12.9	0.01787%	12.9	0.01024%	12.9	0.00947%	12.9	ATRM
51	A0A0F7ZAM7	Hemopexin	*C. adamanteus*	OP	15.7	45.159	0.00482%	6.5	0.00000%	0	0.00196%	7	0.10566%	15.7	ARM
52	T1DKX1	Histone H2A	*C. horridus*	OP	12.5	13.509	0.00873%	12.5	0.00799%	12.5	0.01321%	12.5	0.01577%	12.5	ATRM
53	A0A0K8RVS1	Histone H3	*C. horridus*	OP	11.9	15.229	0.00679%	11.9	0.00507%	11.9	0.00769%	11.9	0.00966%	11.9	ATRM
54	T1D6Q3	Hyaluronidase	*C. horridus*	HYA	28.5	52.503	0.10747%	28.5	0.06123%	28.5	0.06537%	17.4	0.13387%	13.6	ATRM
55	J3SBQ3	Inactive snake venom serine proteinase 13	*C. adamanteus*	SP	30.9	28.577	0.16708%	21.4	0.08562%	22.5	0.00000%	7.6	0.03226%	13	ATM
56	T1DMM6	Kallikrein-CohID-4	*C. oreganus*	SP	43.8	28.204	0.04095%	35.3	0.00000%	21.3	0.00054%	34.9	0.45436%	31	ARM
57	A0A0K8RZ26	L-amino acid oxidase	*C. horridus*	LAAO	68	58.684	1.41758%	57.6	0.95353%	46.9	0.44660%	27.9	0.00673%	52.5	ATRM
58	A0A0K8S046	L-amino acid oxidase	*C. horridus*	LAAO	71.3	58.712	2.96857%	61.2	2.07858%	50.6	1.08936%	25.6	0.03230%	55.8	ATRM
59	K9N7B7	L-amino acid oxidase Cdc18	*C. durissus*	LAAO	63.3	56.827	0.00040%	46.8	0.02149%	46.4	0.00000%	15.7	0.05245%	51.4	ATM
60	P56742	L-amino-acid oxidase	*C. atrox*	LAAO	69.8	58.766	12.81033%	54.7	8.71637%	48.1	4.01148%	23.4	12.03289%	58.5	ATRM
61	T1DP54	LAAO-CohCI-1	*C. oreganus*	LAAO	70	58.662	1.85359%	59.9	2.23285%	50.2	0.63588%	27.5	0.45730%	57.8	ATRM
62	T1DE73	Lectin_alpha-CohCI-2	*C. oreganus*	LEC	43.7	18.197	0.00521%	16.5	0.02411%	32.9	0.04738%	21.5	0.46940%	15.8	ATRM
63	T1E7H7	Lectin_alpha-CohCI-4	*C. oreganus*	LEC	21	17.909	0.04041%	15.3	0.11096%	15.3	0.00000%	5.7	0.07394%	12.7	ATM
64	T1E6W0	Lectin_alpha-CohLL-2	*C. oreganus*	LEC	20.3	18.001	0.01078%	13.9	0.03850%	7	0.01660%	5.1	0.25100%	4.4	ATRM
65	T1E6V8	Lectin_beta-CohLL-2	*C. oreganus*	LEC	27.7	17.227	0.00000%	0	0.00000%	0	0.00453%	5.4	0.14533%	22.3	RM
66	Q8JJ49	Metalloproteinase	*C. molossus*	SVMP	17.9	46.644	0.00165%	14.7	0.00170%	6.5	0.00099%	9.9	2.92596%	9.4	ATRM
67	Q8JJ50	Metalloproteinase	*C. molossus*	SVMP	22	46.893	0.00287%	18.8	0.00137%	8.9	0.00000%	10.1	7.32244%	13.5	ATM
68	A0A0K8RZ12	Metalloproteinase (Type II) 1	*C. horridus*	SVMP	16.1	54.681	0.00345%	10.5	0.00081%	5.6	0.00286%	13.8	0.00124%	5.4	ATRM
69	A0A0K8RZ08	Metalloproteinase (Type II) 4b	*C. horridus*	SVMP	34.9	53.729	1.62955%	21.3	6.07502%	5.2	3.53220%	14.9	3.84243%	21.3	ATRM
70	J3SDW5	Metalloproteinase (Type III) 1a	*C. adamanteus*	SVMP	24	67.329	0.23684%	18	1.62044%	22	0.22667%	13.1	0.06037%	8	ATRM
71	A0A0K8RYU3	Metalloproteinase (Type III) 5b	*C. horridus*	SVMP	49.5	67.914	0.01841%	49.5	0.09931%	40.5	0.09680%	19.2	0.00061%	31	ATRM
72	F8S105	Metalloproteinase 4	*C. adamanteus*	SVMP	20.1	68.017	0.00000%	16.5	0.00000%	11.4	0.00000%	5.9	0.28750%	9	M
73	Q1ZZ79	Metalloproteinase P-I	*C. durissus*	SVMP	44.2	46.856	2.44485%	33.3	1.13032%	19.1	1.80439%	20	11.96786%	26.1	ATRM
74	P12028	Myotoxin-1	*C. oreganus*	OP	46.5	5.061	0.00000%	0	0.00000%	0	0.00000%	0	0.05001%	46.5	M
75	T1E6V6	NGF-CohLL-2	*C. oreganus*	NGF	33.6	27.167	0.12981%	22.4	0.30760%	30.7	0.43405%	23.2	0.13746%	21.6	ATRM
76	A0A0F7Z8F3	Nuclear receptor coactivator 7	*C. adamanteus*	OP	3.1	106.5	0.00000%	0	0.00177%	1	0.00542%	3.1	0.00552%	1	TRM
77	T1D9Q4	Peptidyl-prolyl cis-trans isomerase	*C. horridus*	TBP	12.4	22.82	0.00000%	0	0.00000%	0	0.00070%	12.4	0.00000%	0	R
78	T1D9W7	Peroxiredoxin 4	*C. adamanteus*	OP	17.3	29.545	0.00156%	13.9	0.00137%	12	0.00127%	13.9	0.00172%	7.1	ATRM
79	T1DJS4	Peroxiredoxin-1-like protein	*C. horridus*	OP	13.1	22.326	0.00000%	0	0.00000%	0	0.00000%	0	0.00035%	13.1	M
80	A0A0K8RYS5	Phospholipase A2	*C. horridus*	PLA_2	63.8	15.702	0.10742%	53.6	0.04550%	53.6	0.00794%	44.2	0.55695%	36.2	ATRM
81	B0LSG5	Phospholipase A2	*S. miliarius*	PLA_2	60.1	15.695	3.35383%	60.1	0.63126%	56.5	2.00630%	60.1	0.15125%	50	ATRM

82	B0LSG7	Phospholipase A2	*S. miliarius*	PLA_2	36.2	15.762	0.53174%	34.1	1.21439%	26.8	4.12997%	31.2	0.45992%	21.7	ATRM
83	P0CV89	Phospholipase A2	*C. atrox*	PLA_2	55.7	7.3615	1.32990%	55.7	0.31006%	55.7	0.08543%	55.7	0.16215%	44.3	ATRM
84	J3S0I2	Phospholipase A2 inhibitor 31 kDa subunit-like	*C. adamanteus*	PLA_2	6.3	24.583	0.00000%	0	0.00000%	0	0.00023%	3.2	0.02367%	6.3	RM
85	J3S4V8	Phospholipase A2 inhibitor subunit B	*C. adamanteus*	PLI	21.1	37.253	0.03518%	21.1	0.00501%	11.5	0.00027%	2.7	0.01490%	12.1	ATRM
86	A0A193CHK0	Phospholipase A2_1	*C. molossus*	PLA_2	48.6	15.533	0.00000%	21.7	0.00134%	33.3	0.01072%	33.3	1.62744%	48.6	TRM
87	A0A193CHL0	Phospholipase A2_1	*C. basiliscus*	PLA_2	35.5	15.67	0.00000%	18.1	0.00054%	23.9	0.00071%	18.8	1.70324%	35.5	TRM
88	A0A193CHJ8	Phospholipase A2_2	*C. molossus*	PLA_2	56.2	15.453	4.21272%	46.7	0.01498%	34.3	17.18464%	45.3	9.55447%	51.1	ATRM
89	A0A1W7RB94	Phospholipase B-like	*C. adamanteus*	PLB	47.4	64.109	1.98703%	45.6	1.07517%	44.8	0.48873%	33.8	0.25618%	38.2	ATRM
90	T1E5G4	Plasma protease C1 inhibitor-like protein	*C. horridus*	OP	6.7	56.293	0.00000%	0	0.00034%	4.9	0.00000%	0	0.00084%	1.8	TM
91	T1DJP0	Plastin-3-like protein	*C. horridus*	OP	1.4	70.503	0.00410%	1.4	0.00000%	0	0.01126%	1.4	0.00381%	1.1	ARM
92	A0A0F7Z7R5	Progesterone-induced-blocking factor 1-like	*C. adamanteus*	OP	3.4	89.785	0.00000%	0	0.00306%	2.4	0.00000%	0	0.00000%	1.1	T
93	T1DNE0	Putative serine carboxypeptidase CPVL-like protein	*C. horridus*	TBP	15	55.504	0.02316%	13.2	0.00562%	11.5	0.00588%	8.8	0.01636%	11.5	ATRM
94	J3SF51	Ribonuclease T2-like	*C. adamanteus*	NUC	23.6	30.438	0.00453%	23.6	0.00036%	7.8	0.00064%	17.8	0.00036%	3.1	ATRM
95	A0A2I7YRZ0	Secretory phospholipase A2	*C. lepidus*	PLA_2	63	15.648	1.38984%	63	0.23644%	47.8	3.53540%	40.6	0.06157%	42	ATRM
96	A0A2I7YRZ4	Secretory phospholipase A2	*C. lepidus*	PLA_2	72.5	15.736	4.38932%	72.5	9.61670%	72.5	17.75787%	47.8	6.60990%	52.9	ATRM
97	A0A2I7YS22	Secretory phospholipase A2	*C. lepidus*	PLA_2	71	15.744	0.34707%	71	0.92068%	68.8	0.00000%	24.6	0.00022%	44.2	ATM
98	A0A2I7YS24	Serine endopeptidase	*C. atrox*	SP	53.1	27.719	1.53675%	53.1	0.61489%	52.7	0.77937%	29.1	0.07303%	39.9	ATRM
99	A0A2I7YS26	Serine endopeptidase	*C. atrox*	SP	28.3	28.086	0.01824%	28.3	0.00495%	18.6	0.00930%	28.3	0.00000%	20.2	ATR
100	A0A2I7YS33	Serine endopeptidase	*C. cerastes*	SP	63.9	29.045	0.00018%	62	0.00146%	63.9	0.00000%	18.3	0.00000%	38.4	AT
101	A0A2I7YS37	Serine endopeptidase	*C. cerastes*	SP	18.6	27.785	0.27178%	18.6	0.04717%	18.2	0.00734%	14	0.00000%	6.2	ATR
102	A0A2I7YS41	Serine endopeptidase	*C. atrox*	SP	36.1	29.122	0.00152%	21.7	0.00000%	20.5	0.00000%	11	0.15555%	26.6	AM
103	A0A2I7YS44	Serine endopeptidase	*C. cerastes*	SP	60.1	28.015	0.07471%	60.1	0.02684%	60.1	0.03689%	24.4	0.00014%	43.8	ATRM
104	A0A2I7YS46	Serine endopeptidase	*C. lepidus*	SP	52.3	28.274	1.84117%	52.3	0.69647%	44.6	0.34233%	23.6	0.11550%	26.4	ATRM
105	A0A2I7YS48	Serine endopeptidase	*C. lepidus*	SP	56.6	28.008	0.72283%	56.6	0.17473%	56.2	0.04660%	29.8	0.00089%	24	ATRM
106	A0A2I7YS49	Serine endopeptidase	*C. lepidus*	SP	37.1	28.251	4.03480%	37.1	1.21273%	37.1	1.92534%	24.7	0.00293%	28.2	ATRM
107	A0A2I7YS51	Serine endopeptidase	*C. lepidus*	SP	21.1	27.735	0.00265%	21.1	0.00000%	7.4	0.00000%	7.4	0.00000%	7.4	A
108	A0A2I7YS53	Serine endopeptidase	*C. lepidus*	SP	30.2	27.559	0.26421%	21.7	0.00791%	30.2	0.00000%	17.4	0.00125%	26	ATM
109	A0A2I7YS56	Serine endopeptidase	*C. lepidus*	SP	35.9	28.914	0.58040%	35.9	0.23684%	35.9	0.00951%	27.4	0.01938%	27.4	ATRM
110	A0A2I7YS61	Serine endopeptidase	*C. lepidus*	SP	69.1	29.264	0.55495%	68.3	2.16708%	51.1	0.14226%	21.4	0.00916%	15.3	ATRM
111	A0A2I7YS62	Serine endopeptidase	*C. molossus*	SP	58.9	28.466	0.00000%	52.3	0.00000%	49.6	0.00000%	22.1	0.06192%	27.1	M
112	A0A2I7YS63	Serine endopeptidase	*C. lepidus*	SP	75.3	29.082	1.34118%	65.8	1.81920%	61.6	0.00712%	18.6	1.71006%	38.8	ATRM
113	A0A2I7YS65	Serine endopeptidase	*C. lepidus*	SP	59.7	28.469	1.77585%	59.7	0.58605%	59.7	0.68288%	32.2	0.01796%	20.5	ATRM
114	A0A2I7YS67	Serine endopeptidase	*C. mitchellii*	SP	20.4	28.805	0.00000%	7.7	0.00000%	7.7	0.00000%	7.7	0.00916%	20.4	M
115	A0A2I7YS75	Serine endopeptidase	*C. mitchellii*	SP	73.5	28.549	2.77720%	69.2	1.00634%	67.7	0.33932%	15.4	0.99912%	39.2	ATRM
116	A0A2I7YS76	Serine endopeptidase	*C. lepidus*	SP	67.3	28.041	0.08012%	67.3	0.04825%	66.1	0.00305%	16	0.00042%	38.1	ATRM

117	A0A2I7YS77	Serine endopeptidase	*C. molossus*	SP	37.3	28.701	0.02151%	33.8	0.03230%	37.3	0.00051%	18.8	0.00545%	26.5	ATRM
118	A0A2I7YS83	Serine endopeptidase	*C. molossus*	SP	16.4	28.342	0.00000%	12.2	0.00000%	11.8	0.00000%	11.8	0.01538%	16	M
119	A0A2I7YS84	Serine endopeptidase	*C. molossus*	SP	72.1	27.939	1.60872%	64	0.57903%	36.8	0.67498%	16.7	1.10442%	57	ATRM
120	A0A2I7YS88	Serine endopeptidase	*C. scutulatus*	SP	20.6	28.312	0.20631%	20.6	0.33802%	9.7	0.02000%	6.6	0.00037%	9.3	ATRM
121	A0A2I7YS89	Serine endopeptidase	*C. scutulatus*	SP	52.1	27.742	0.38783%	49	0.20907%	49	0.17261%	17.5	0.03166%	28	ATRM
122	A0A2I7YS95	Serine endopeptidase	*C. molossus*	SP	74.7	28.04	0.67148%	74.7	0.12903%	71.2	0.15348%	26.5	0.19250%	30	ATRM
123	A0A2I7YSA2	Serine endopeptidase	*C. scutulatus*	SP	62.4	29.043	0.02545%	44.9	0.01512%	61.6	0.00000%	14.8	0.00088%	38.8	ATM
124	A0A2I7YSA9	Serine endopeptidase	*C. tigris*	SP	17.9	29.547	0.01210%	9.5	0.02660%	9.5	0.00000%	3.4	0.00406%	11.8	ATM
125	A0A0K8RYM8	Serine proteinase 7	*C. horridus*	SP	56.5	28.646	0.00765%	52.3	0.00048%	50.8	0.00000%	15.4	0.00000%	35.8	AT
126	T1E3B5	Serine proteinase 7	*C. horridus*	SP	40.7	28.152	0.56310%	40.7	0.08188%	35.7	0.16050%	40.3	0.36598%	32.2	ATRM
127	T1DJX6	Serine proteinase 9	*C. horridus*	SP	39.7	28.573	0.00374%	39.7	0.00363%	31.1	0.00000%	22.6	0.00000%	22.6	AT
128	T1DIT7	Serpin B6-like protein	*C. horridus*	OP	5.3	43.058	0.00000%	0	0.00000%	0	0.00000%	0	0.01128%	5.3	M
129	P81509	Snaclec CHH-B subunit beta	*C. horridus*	CTL	64.1	13.888	0.01297%	30.8	0.07221%	18.8	0.13806%	20.5	0.33402%	53	ATRM
130	F8S0Z7	Snake venom 5-nucleotidase	*C. adamanteus*	NUC	49.1	64.681	0.42605%	41.7	0.34967%	43.9	0.42220%	31.3	1.10884%	33.3	ATRM
131	Q8JJ51	Snake venom metalloproteinase	*C. molossus*	SVMP	21.5	46.908	0.18112%	21.5	0.09050%	8.9	0.05481%	15.2	0.05989%	9.4	ATRM
132	J3RY78	Snake venom metalloproteinase (Type II) 7	*C. adamanteus*	SVMP	24	53.888	0.15260%	17.7	0.09614%	9.6	0.01449%	15.4	0.11602%	16.7	ATRM
133	T1DJY5	Snake venom metalloproteinase (Type III)	*C. horridus*	SVMP	22.6	68.429	0.07673%	20.1	0.11123%	13.7	0.26081%	17.2	0.05649%	9.8	ATRM
134	J3S831	Snake venom metalloproteinase (Type III) 5	*C. adamanteus*	SVMP	27.8	68.434	0.23950%	26.2	0.11278%	10	0.01120%	20.7	0.00291%	10.4	ATRM
135	J3RY90	Snake venom metalloproteinase (Type III) 6	*C. adamanteus*	SVMP	28	69.652	2.55938%	27.5	4.90322%	18	2.53856%	15.6	0.03762%	13.5	ATRM
136	Q90391	Snake venom metalloproteinase atrolysin-B	*C. atrox*	SVMP	47.1	46.805	0.96538%	32.9	0.01540%	14.3	0.26442%	20.8	0.64321%	26.6	ATRM
137	Q90392	Snake venom metalloproteinase atrolysin-C	*C. atrox*	SVMP	34.1	46.768	5.87434%	30.2	4.36705%	14.3	10.17922%	16.7	0.37453%	16.7	ATRM
138	Q9PSN7	Snake venom metalloproteinase HT-1	*C. ruber*	SVMP	45.8	23.601	0.08328%	14.8	0.04721%	25.5	0.06257%	13.4	0.05504%	35.2	ATRM
139	P20897	Snake venom metalloproteinase HT-2	*C. ruber*	SVMP	30.7	23.32	0.27472%	22.3	2.08100%	22.3	0.04258%	18.8	0.14879%	22.3	ATRM
140	Q2QA04	Snake venom serine protease	*C. durissus*	SP	34.7	28.27	0.00000%	24.8	0.00000%	24.4	0.00000%	16	0.39728%	26	M
141	Q8QHK2	Snake venom serine protease catroxase-2	*C. atrox*	SP	72.1	27.909	0.21188%	64	0.08440%	44.2	0.21468%	24	0.00078%	49.6	ATRM
142	J3S3W5	Snake venom serine proteinase	*C. adamanteus*	SP	53.8	29.456	1.33500%	45.4	0.45213%	32.4	0.00000%	9.5	0.02855%	22.5	ATM
143	J3RYA3	Snake venom serine proteinase 9	*C. adamanteus*	SP	66	29.53	0.14470%	48.9	0.37646%	48.1	0.00000%	13	0.14590%	22.5	ATM

144	C0K3N3	Snake venom vascular endothelial growth factor toxin cratrin	*C. atrox*	VEGF	20.1	16.126	0.87448%	17.4	0.92119%	20.1	1.44373%	17.4	0.03492%	5.6	ATRM
145	T1DEB4	SVMP-CohPH-2	*C. oreganus*	SVMP	22.8	49.124	0.03606%	20	0.03380%	20	0.00877%	12.5	0.00000%	4.8	ATR
146	B0FXM2	Thrombin-like enzyme gyroxin B1.4	*C. durissus*	SP	32.1	29.261	0.00000%	16.4	0.00073%	15.6	0.00000%	3.4	0.00000%	6.9	T
147	A0A0F7Z911	Transcriptional regulator ATRX-like	*C. adamanteus*	OP	0.8	267.04	0.00371%	0.5	0.00266%	0.5	0.00442%	0.5	0.01273%	0.8	ATRM
148	A0A0F7Z1V6	Transferrin	*C. adamanteus*	OP	16.7	77.737	0.00275%	10.8	0.00019%	1.3	0.00430%	7.5	0.14873%	14.1	ATRM
149	T1D7J0	Tropomyosin alpha-4 chain-like protein	*C. horridus*	OP	8.4	28.864	0.00188%	8.4	0.00201%	8.4	0.00373%	4.4	0.00076%	8.4	ATRM
150	T1E4B0	Tubulin alpha chain	*C. horridus*	OP	22	49.895	0.00473%	22	0.00258%	12.5	0.00693%	22	0.00475%	18	ATRM
151	T1DHR5	Tubulin beta chain	*C. horridus*	OP	16.9	49.67	0.00082%	4.5	0.00051%	4.5	0.00451%	14.6	0.00264%	11	ATRM
152	T1E3W8	Venom factor	*C. horridus*	OP	3.5	184.97	0.00329%	3.5	0.00000%	0	0.00103%	2.7	0.00000%	0	AR
153	A0A0K8RSA 1	Vespryn 1b	*C. horridus*	OP	12.9	20.782	0.00000%	0	0.00000%	0	0.00106%	12.9	0.02089%	12.9	RM
154	J3SFM5	Vimentin	*C. adamanteus*	OP	5.7	52.671	0.00081%	5.7	0.00025%	2.2	0.00039%	2.2	0.00152%	4.2	ATRM
155	C9E1R7	Zinc metalloproteinase-disintegrin VMP-II	*C. atrox*	DIS	32.1	54.736	0.34208%	28.4	0.26899%	14.8	0.01312%	14.4	0.33978%	20.2	ATRM
156	J9Z332	Zinc metalloproteinase-disintegrin VMP-II	*C. adamanteus*	SVMP	25.6	54.822	0.01350%	22.1	0.00599%	16.2	0.00204%	10.5	0.00734%	18.4	ATRM
157	J3S830	Zinc metalloproteinase-disintegrin-like 3a	*C. adamanteus*	SVMP	19.7	68.84	0.21194%	19.7	0.59193%	11.1	0.19362%	11.8	0.02224%	8.2	ATRM
158	F8S108	Zinc metalloproteinase-disintegrin-like 4a	*C. adamanteus*	SVMP	57.5	67.909	3.82081%	57.5	2.89428%	41.8	0.22951%	21.8	0.71376%	29.8	ATRM
159	J3SDW8	Zinc metalloproteinase-disintegrin-like 8	*C. adamanteus*	SVMP	30.1	67.8	0.31345%	22.2	1.15504%	22.4	0.00068%	5.9	0.17304%	10.3	ATRM
160	Q92043	Zinc metalloproteinase-disintegrin-like atrolysin-A	*C. atrox*	SVMP	23.2	46.879	0.02462%	17.9	0.04706%	17.9	0.00871%	19.8	0.01479%	14.3	ATRM
161	C5H5D1	Zinc metalloproteinase-disintegrin-like crotastatin	*C. durissus*	DIS	47.4	46.164	0.01235%	45.5	0.00422%	45.5	0.00260%	25.6	1.00888%	41.6	ATRM
162	Q9DGB9	Zinc metalloproteinase-disintegrin-like VAP1	*C. atrox*	SVMP	53.1	67.96	0.01270%	51.5	0.03144%	44.1	0.00200%	22.3	0.00149%	31.3	ATRM
163	A4PBQ9	Zinc metalloproteinase-disintegrin-like VAP2A	*C. atrox*	SVMP	36.7	68.034	7.39720%	35.3	14.29149%	25.2	4.64929%	28.8	17.19499 %	27.3	ATRM
164	P34182	Zinc metalloproteinase/disintegrin	*C. atrox*	SVMP	26.4	53.637	0.56178%	12.8	1.65655%	12.8	4.12277%	11.1	1.34055%	15.9	ATRM
165	C9E1R9	Zinc metalloproteinase/disintegrin VMP-II	*C. viridis*	DIS	21.5	53.813	0.14730%	21.3	0.47149%	15.3	1.94874%	12.1	0.00080%	7.5	ATRM
